# Chromothripsis detection with multiple myeloma patients based on deep graph learning

**DOI:** 10.1093/bioinformatics/btad422

**Published:** 2023-07-03

**Authors:** Jixiang Yu, Nanjun Chen, Zetian Zheng, Ming Gao, Ning Liang, Ka-Chun Wong

**Affiliations:** Department of Computer Science, City University of Hong Kong, Kowloon, 999077, Hong Kong; Department of Computer Science, City University of Hong Kong, Kowloon, 999077, Hong Kong; Department of Computer Science, City University of Hong Kong, Kowloon, 999077, Hong Kong; School of Management Science and Engineering, Dongbei University of Finance and Economics, Dalian 116025, China; University of Michigan, Ann Arbor, MI 48105, United States; Department of Computer Science, City University of Hong Kong, Kowloon, 999077, Hong Kong; Shenzhen Research Institute, City University of Hong Kong, Shenzhen 518057, China; Hong Kong Institute for Data Science, City University of Hong Kong, Kowloon, 999077, Hong Kong

## Abstract

**Motivation:**

Chromothripsis, associated with poor clinical outcomes, is prognostically vital in multiple myeloma. The catastrophic event is reported to be detectable prior to the progression of multiple myeloma. As a result, chromothripsis detection can contribute to risk estimation and early treatment guidelines for multiple myeloma patients. However, manual diagnosis remains the gold standard approach to detect chromothripsis events with the whole-genome sequencing technology to retrieve both copy number variation (CNV) and structural variation data. Meanwhile, CNV data are much easier to obtain than structural variation data. Hence, in order to reduce the reliance on human experts’ efforts and structural variation data extraction, it is necessary to establish a reliable and accurate chromothripsis detection method based on CNV data.

**Results:**

To address those issues, we propose a method to detect chromothripsis solely based on CNV data. With the help of structure learning, the intrinsic relationship-directed acyclic graph of CNV features is inferred to derive a CNV embedding graph (i.e. CNV-DAG). Subsequently, a neural network based on Graph Transformer, local feature extraction, and non-linear feature interaction, is proposed with the embedding graph as the input to distinguish whether the chromothripsis event occurs. Ablation experiments, clustering, and feature importance analysis are also conducted to enable the proposed model to be explained by capturing mechanistic insights.

**Availability and implementation:**

The source code and data are freely available at https://github.com/luvyfdawnYu/CNV_chromothripsis.

## 1 Introduction

Multiple myeloma (MM) is a type of bone marrow cancer that may progress toward aggressive states in extramedullary locations within short periods (Magrangeas *et al.* 2011, Neuse *et al.* 2020). Gene rearrangements and chromothripsis were identified in the genomic sequences of a proportion of MM patients (Magrangeas *et al.* 2011). More importantly, patients with catastrophic chromothripsis are often associated with poor clinical outcomes (Ashby *et al.* 2018, 2022). In contrast, clinically stable myeloma (i.e. non-progressive MM) tends to progress later in the patients’ life compared to progressive MM patients (Oben *et al.* 2021). In such cases, chromothripsis and templated insertion were not observed in the genomes of patients (Oben *et al.* 2021). Meanwhile, Maclachlan *et al.* (2021) suggested that chromothripsis can be detected in advance years before the progression of MM. Previous studies have shown that chromothripsis is a potent prognostic factor of progressive MM (Ashby *et al.* 2019, Maura *et al.* 2021). Moreover, detecting chromothripsis before the progression of MM gives a chance to formulate a personalized therapy for MM patients (Luijten *et al.* 2018).

Chromothripsis is characterized by up to thousands of cluster chromosomal rearrangements that occur simultaneously (Leibowitz *et al.* 2015, Zhang *et al.* 2015, Cortés-Ciriano *et al.* 2020). The incidence of chromothripsis is particularly prevalent in bone cancers (Kloosterman and Cuppen 2013) and is linked to poor prognosis within a wide range of bone cancer patients (Fontana *et al.* 2018, Voronina *et al.* 2020, Kolmar *et al.* 2022). Chromothripsis event identification has become a routine part of cancer research (Yang *et al.* 2016). Highly accurate chromothripsis detection is clinically significant since patients may benefit from regular screening and careful treatment strategies (Maher and Wilson 2012, Luijten *et al.* 2018). Traditionally, chromothripsis detection depends on a combination of whole-genome sequencing (WGS) with structural variation (SV) and copy number variation (CNV) data (Cortés-Ciriano *et al.* 2020). However, domain experts’ manual diagnosis still remains the gold standard approach (Maclachlan *et al.* 2021). Furthermore, recent studies of SVs using orthogonal technologies have shown that SVs are the least well-characterized type of genetic variant, with many basic questions, such as the average number of SVs per sample or sequence biases contributing to their formation, still not completely resolved (Denti *et al.* 2022). Although SVs are more significant in chromothripsis detection, the data extraction is still very difficult. Maclachlan *et al.* (2021) first explored the relationship between CNV signatures and chromothripsis based on the WGS data from 752 newly diagnosed multiple myeloma (NDMM) patients and found them highly correlated. They leveraged the CNV feature construction method proposed by Macintyre *et al.* (2018) to obtain the CNV features. Considering the abnormal changes of genome-wide copy number (CN) in different aspects, six types of fundamental CNV features were obtained, namely, (i) the number of breakpoints per 10 Mb, (ii) absolute CN of segments, (iii) the difference in CN between adjacent segments, (iv) number of breakpoints per chromosome arm, (v) lengths of oscillating CN segment chains, and (vi) the size of segments. Subsequently, they leveraged a mixture model called hierarchical Dirichlet processes to obtain the CNV signatures. Eventually, chromothripsis is detected using a logistic regression model with CNV signatures as the input. Combined with SV signatures, the performance of their method exceeds the state-of-the-art chromothripsis detection method ShatterSeek (Cortés-Ciriano *et al.* 2020). Most importantly, they claimed the high feasibility of detecting chromothripsis with its adverse prognostic impact by using CNV signatures alone, without requiring specific SV assessment (Maclachlan *et al.* 2021).

Deep learning (DL) algorithms powered by advanced computing units have outperformed humans in visual tasks, such as playing Atari games (Mnih *et al.* 2015) and strategic games, such as Go (Silver *et al.* 2018). DL has also outperformed domain experts on biological and chemical datasets (Jumper *et al.* 2021) and has a considerable chance of becoming one of the candidates to replace the gold standard method in this field (Farswan *et al.* 2021, Ganini *et al.* 2021). For instance, Glessner *et al.* (2021) designed a two-branch neural network that combines a convolutional neural network (CNN) and a fully connected network and further used it to detect CNV. Its accuracy surpasses other machine-learning methods (Glessner *et al.* 2021). In work by AlShibli and Mathkour (2019), a CNN-based neural network was designed to classify cancers using CNV data (AlShibli and Mathkour 2019). SVision is another novel DL architecture to detect SV; it introduced a sequence-to-image coding schema, alleviating the complexity of detecting previously uncharacterized complex rearrangements (Lin *et al.* 2022). Through the literature review, a carefully designed DL algorithm is considered to have the potential to detect chromothripsis by leveraging CNV features; and its effect is worth exploring (Cortés-Ciriano *et al.* 2022).

Current chromothripsis detection methods need both CNV and SV data (Cortés-Ciriano *et al.* 2020). From a technical perspective, however, obtaining SV is harder than CNV with more computational complexity since high-confident SV detection needs the integration of multiple algorithms and sequencing technologies, accompanied with a series of quality filters to detect complicated SVs, such as long-range translocations (van Belzen *et al.* 2021). Although it is possible to obtain CNV and SV simultaneously, more efforts are required to get them both than obtaining CNV only (Mahmoud *et al.* 2019, van Belzen *et al.* 2021). On the other hand, the source to obtain CNV is more flexible than SV from a clinical diagnosis perspective. CNV can also be obtained via whole-exome sequencing (WES), which is much cheaper and more practical than WGS in actual clinical use, in MM research (Shen and Seshan 2016, Maclachlan *et al.* 2021). There are also some toolkits to generate CNV profile from WES data (Krumm *et al.* 2012, Packer *et al.* 2016, Babadi *et al.* 2022). In contrast, SVs are difficult to detect using WES data (Fadaie *et al.* 2021, Hou *et al.* 2022), and getting large segments of SV through short read sequences is equally challenging. At the same time, the DL application with CNV to detect chromothripsis remains speculative. Therefore, it is necessary to develop a reliable and accurate DL-based chromothripsis detection method solely based on CNV data to reduce the reliance on human experts’ efforts and the difficulty of data extraction and to be align with practical clinical diagnosis scenario.

Based on the CNV features of NDMM patients (n=752) by Maclachlan *et al.* (2021), we propose and design a DL algorithm to facilitate accurate chromothripsis detection. In order to make full use of the intrinsic relationships between multiple CNV features, the state-of-the-art structure learning method NOTEARS (Zheng *et al.* 2018) is used to conduct causal inferences between CNV features and derive an embedding directed acyclic graph (DAG) of the six types of CNV features (i.e. CNV-DAG). Subsequently, we propose the Graph Embedding CNV Network (GECNVNet), with the CNV-DAG as the input to detect chromothripsis. Inside GECNVNet, the Graph Transformer (Shi *et al.* 2020) with dot product attention is leveraged for the graph feature embedding learning based on the inferred CNV-DAG. Meanwhile, we design the local feature extraction module and the non-linear feature interaction module in GECNVNet, combined with second-order bilinear pooling for further feature representation learning. Experimental results on the real dataset listed in Table 1 reflect that our method can effectively learn the latent graph representations and significantly outperform other baseline methods. Additionally, the performance of GECNVNet can be pushed further when SV is given. Moreover, in order to demonstrate chromothripsis detected by GECNVNet are predictable of poor clinical outcome, progression-free survival (PFS), and overall survival (OS) analysis are also conducted. The results demonstrate that the proposed chromothripsis detection methodology (GECNVNet) has the feasibility of distinguishing high-risk from low-risk MM patients, consistent with the existing belief that chromothripsis is a strong prognostic factor of progressive MM.

**Table 1.** The experimental results of AUPRC on five different splits.^a^

Data input	Method	Split1	Split2	Split3	Split4	Split5	AUPRC (AVG ± SD)
CNV signature	LASSO (Maclachlan *et al.* 2021)*	0.8648	0.8016	0.8116	0.7728	0.7498	0.8001 ± 0.044
	RIDGE*	0.8599	0.7954	0.8099	0.7595	0.7478	0.7945 ± 0.045
	SVM(RBF)*	0.7623	0.7030	0.5770	0.7136	0.6842	0.6880 ± 0.068
	SVM(linear)*	0.8484	0.8036	0.7990	0.7586	0.7661	0.7951 ± 0.036
	RF*	0.7751	0.7707	0.7164	0.7342	0.7177	0.7428 ± 0.028
	XGBoost*	0.7464	0.7739	0.7294	0.7610	0.7822	0.7586 ± 0.021
CNV feature	LASSO*	0.7114	0.7798	0.7930	0.7583	0.7348	0.7555 ± 0.033
	RIDGE*	0.6953	0.7756	0.7857	0.7504	0.7246	0.7463 ± 0.037
	SVM(RBF)*	0.8168	0.7422	0.7697	0.7852	0.6927	0.7613 ± 0.047
	SVM(linear)*	0.6796	0.7784	0.7673	0.7725	0.7220	0.7440 ± 0.042
	RF*	0.8668	0.7796	0.8057	0.8112	0.7902	0.8107 ± 0.034
	XGBoost*	0.8344	0.7581	0.7868	0.7880	0.8066	0.7949 ± 0.028
	MLP*	0.8298	0.7775	0.7981	0.7205	0.7811	0.7814 ± 0.040
	CNN*	0.8107	0.7675	0.7890	0.7828	0.7052	0.7710 ± 0.040
	Graph Transformer (Shi *et al.* 2020)*	0.8553	0.7716	0.7860	0.7538	0.7732	0.7880 ± 0.039
	GCN (Kipf and Welling 2016)*	0.8304	0.7291	0.7023	0.7517	0.7650	0.7557 ± 0.048
	GAT (Veličković *et al.* 2017)*	0.8500	0.7191	0.7285	0.7511	0.7835	0.7664 ± 0.053
	Transformer (Vaswani *et al.* 2017)*	0.8127	0.7935	0.7560	0.7807	0.7429	0.7772 ± 0.028
	GECNVNet(Ours)	**0.8787**	**0.8176**	**0.8202**	**0.8211**	**0.8171**	**0.8309 ** ± **0.027**

a CNV signature: the data input is processed using HDP mixture model as Maclachlan *et al.* (2021). CNV feature: the data input is the original 28 categories CNV feature, SVM(RBF): SVM with Gaussian Radial Basis function kernel, SVM(linear): SVM with linear function kernel, MLP: a four-layer multi-layer perceptron neural network, CNN: a four-layer convolutional neural network, Graph Transformer: a four-layer Graph Transformer neural netowork, GCN: a four-layer graph convolutional neural network, GAT: a four-layer graph attention neural network, Transformer: a four-layer Transformer encoder neural network. Bold values: the best performance.

* *P*-value of the Wilcoxon signed-rank test of AUPRC < .05.

Additionally, ablation experiments are carried out on a group of edges from the source vertex to its adjacent nodes in the CNV-DAG to explore how the inferred CNV-DAG structure contributes to the overall neural network model performance. In particular, we attribute it to the observation that some of the inferred edges in the derived CNV-DAG structure are significant for chromothripsis detection and model interpretation. Meanwhile, samples are clustered by the *K*-means algorithm according to the features of the source vertex in the CNV-DAG. For samples in different clusters, the features of the source vertex’s adjacent nodes demonstrate various patterns, which verify that the edges derived by structure learning are reasonable. Moreover, a feature importance analysis is conducted to reveal the underlying genomic insights. The large number of long contiguous CN segments oscillating between two CN states is found as a potent signal associated with chromothripsis occurrence.

## 2 Materials and methods

### 2.1 Dataset

The dataset released by Maclachlan *et al.* (2021) is adopted in this study. In that dataset, genome-wide somatic CN profiles were generated from 752 NDMM patients with low-coverage long-insert WGS (median 4–8×) from the CoMMpass study (Keats *et al.* 2013). Based on the CN profiles generated from 752 NDMM patients’ WGS data, Maclachlan *et al.* (2021) defined 28 features across 6 types of CNV features, where they use the “mclust” package in R that provides a method for clustering and analysis using Gaussian finite mixture model (Scrucca *et al.* 2016). More specifically, six types of CNV features are counted for each sample. According to the distribution of all samples’ features, the optimum number of categories and several thresholds are obtained using the “mclust” package for each CN feature via selecting the largest clustering density. Divided by these thresholds, 28 CNV features are established. The thresholds of those CNV features can be found in the Supplementary Material. The PFS and OS information are also reported in the dataset. The chromothripsis occurrence is labeled manually by Maclachlan *et al.* (2021). According to them, chromothripsis was observed at least once in 24% of the whole dataset.

### 2.2 Overview of the proposed method to detect chromothripsis

**Figure 1.**
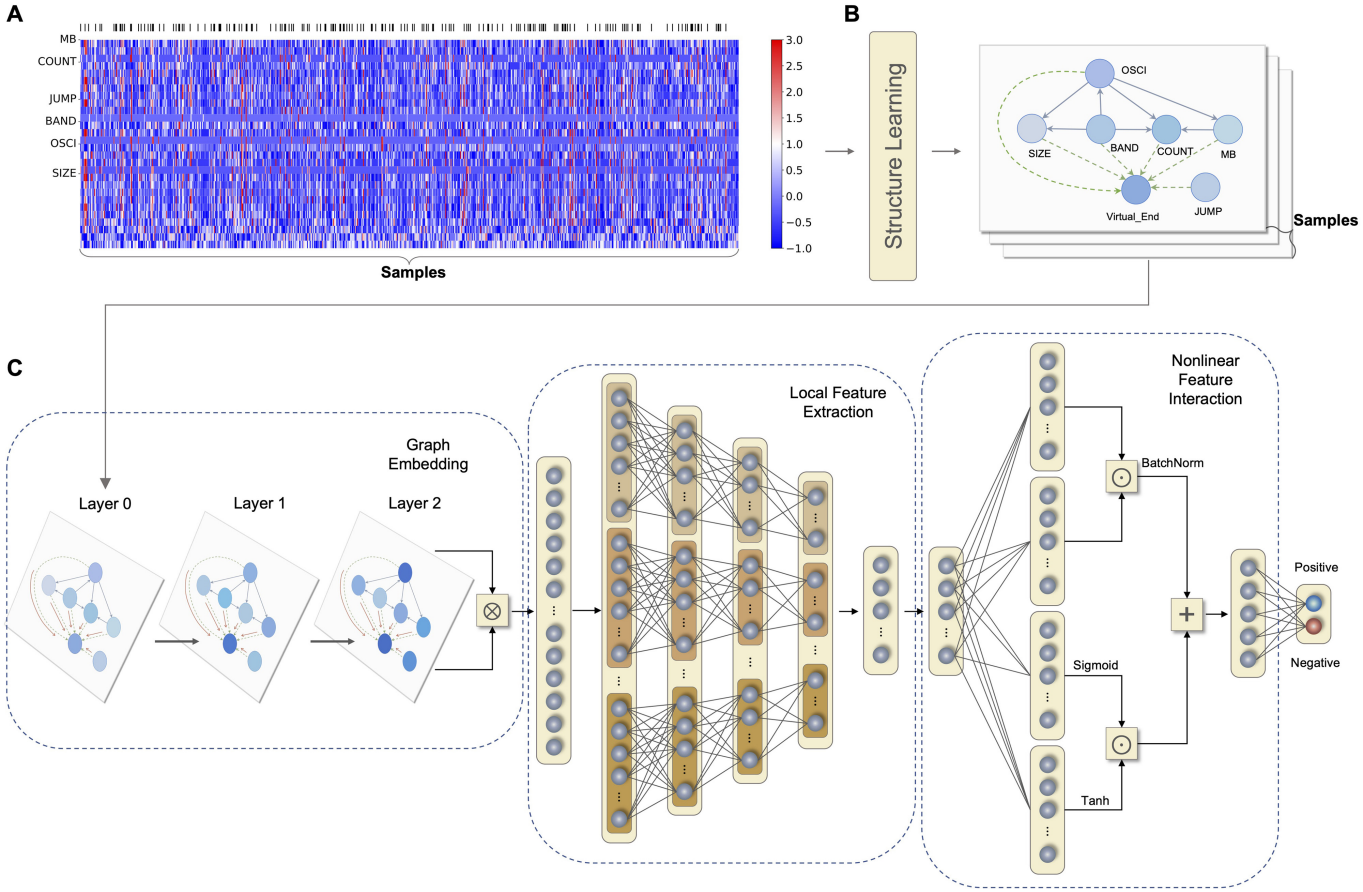
The overview of the proposed chromothripsis detection process. (A) A heatmap of CNV features. Twenty-eight categories of CNV features are demonstrated after standardization. The presence of chromothripsis is annotated in black in the top bar. (B) The feature embedding graph of CNV features established using structure learning. Virtual edges are drawn in dash lines. (C) The overall architecture of GECNVNet, which contains a graph embedding module, a local feature extraction module, a non-linear feature interaction module, and a classifier

As shown in Fig. 1, the CNV features are fed into the GECNVNet to be classified. Specifically, the CNV features are standardized. At the same time, an embedding graph is established and derived by structure learning. Subsequently, the CNV feature vector of each NDMM patient is formulated as the embedding graph, where the node features are padded with zeros. The graphs are fed into GECNVNet to be computed for detection probabilities, which are further used to divide the patients into two groups (i.e. high-risk and low-risk) for their clinical outcome prediction. The abbreviations of the six types of CNV features are listed as follows.

- MB: the number of CN breakpoints per 10 Mb.
- COUNT: absolute CN of segments.
- JUMP: the difference in CN between adjacent segments.
- BAND: number of CN breakpoints per chromosome arm.
- OSCI: lengths of oscillating CN segment chains.
- SIZE: the size of CN segments.

### 2.3 Structure learning to construct the CNV-DAG

DAGs are intuitive representations with extensive applications in causal inference (Spirtes *et al.* 2000). The task of structure learning refers to learning DAG structure from data (Vowels *et al.* 2022). Specifically, let $X\in R^{n\times d}$ be a matrix with *n* independent and identically distributed (i.i.d.) representations of vector $X\in R^{d}$, the structure learning algorithms are considered to find the underlying causal graphical model $\mathrm{CGM}(P_{X},G)$ and output the causal graph G including bivariate data with causal directions, where $P_{X}$ denotes the joint distribution of *X* (Guo *et al.* 2020, Vowels *et al.* 2022). A causal graph G=(V,E) is a DAG that describes the dependencies between variables, where V denotes the node set and E denotes the edge set. In a causal graph, each node represents a variable. A directed edge x→y denotes the causal effect of *x* on *y* (Guo *et al.* 2020).

The use of CNV features in genomic analysis has become increasingly popular in recent years, as these features provide valuable information about the genetic makeup of samples (Drews *et al.* 2022, Steele *et al.* 2022). In our case, some of the CNV features are highly correlated with each other, e.g. BAND and MB, making it difficult to determine their individual contributions to MM. Therefore, we aim to leverage structure learning methods to generate a DAG that captures the learned relationships between CNV features. By doing so, we can also understand the underlying mechanisms behind MM. To achieve this goal, we employ the state-of-the-art structure learning method NOTEARS (Zheng *et al.* 2018). This method has been shown to be effective in learning DAGs from tabular data with i.i.d. samples, making it well-suited for our analysis of the 28 categories of CNV features in a visually explainable manner. A visualization of the learned causal DAG can be found in the Supplementary Material.

Subsequently, the six types of CNV features are modeled as six nodes, and the edges obtained from the NOTEARS algorithm are aggregated to derive a CNV-DAG. Here, we also assume that all six types of CNV features contribute to chromothripsis. Hence, a virtual node that representing chromothripsis is added in the CNV-DAG, in which virtual edges from all feature nodes to the virtual node are also inserted. To enhance the message passing mechanism of the GNN in later process, self-loops are added in the graph. Eventually, a feature embedding graph (i.e. CNV-DAG) with seven nodes is established, as shown in Fig. 1B. The CNV features of each sample are modeled as the feature embedding graph and subsequently fed into the neural network shown in Fig. 1C.

### 2.4 GECNVNet architecture

The overall neural network architecture consists of three main modules: graph embedding module, local feature extraction module, and non-linear feature interaction module. The proposed neural network architecture is lightweight and easy-to-replicate with only 0.1 M parameters.

#### 2.4.1 Graph embedding module

Let G(V,E) denote the modeled CNV-DAG of one sample, $X=[x_{1},x_{2},\ldots ,x_{7}]$ denote the node features of *G*, where $x_{i}$ denote the *i*-th type of CN feature. The node features are required to be projected to high-dimensional spaces for further feature representation learning. To this end, the Graph Transformer proposed by Shi *et al.* (2020) is leveraged for feature embedding. Given the node feature $x_{i}$ in *G*, the calculation of the Graph Transformer layer is defined as: where the attention coefficient $\alpha_{i,j}$ is computed via dot product attention: where $W_{1}$, $W_{2}$, $W_{3}$, and $W_{4}$ are different weights matrices of the Graph Transformer layer, N(i) is the neighborhood set of node *i*, and *d* is the dimension of $x_{i}$ and $x_{j}$. Given the node feature matrix $X\in R^{7\times d_{0}}$, the abstract of the calculation in the Graph Transformer Layers can be written as: where *l* denotes the iteration times of X, and 0≤l≤L. When l=0, $X^{0}:=X$, and when l=L, $X^{L}\in R^{7\times d_{L}}$, in which $d_{L}$ denotes the dimension of the node feature after *L* iterations. Subsequently, the bilinear pooling operation (Lin *et al.* 2015) is performed to obtain the second-order feature self-interactions. The calculation of bilinear pooling is represented as: where $X_{p}\in R^{d_{L}\times d_{L}}$. Then, the $X_{p}$ is reshaped to an 1D vector for feature extraction.


$$x_{i}^{\prime }:=W_{1}x_{i}+\sum_{j\in N(i)}\alpha_{i,j}W_{2}x_{j},$$

$$\alpha_{i,j}:=\text{softmax}(\frac{{\left(W_{3}x_{i}\right)}^{T}(W_{4}x_{j})}{\sqrt{d}}),$$

$$X^{l+1}:=\text{BatchNorm}(\text{GraphTransformer}(X^{l})),$$

$$X_{p}:=\text{ReLU}({\left(X^{L}\right)}^{T}X^{L}),$$

#### 2.4.2 Local feature extraction module

From the perspective of the entire network architecture, the local feature extraction module contains four locally connected 1D layers, which is designed based on fully connected network. Each layer consists of *n* independent fully connected networks. The input of the layers is subdivided to *n* regions corresponding to the layers for feature extraction, which can be defined as $X_{e}^{0}:=\{X_{p}^{0}(1),X_{p}^{0}(2),\ldots ,X_{p}^{0}(n)\}$. As such, the calculation of the local feature extraction module can be defined as: where $X_{e}^{k}(1),X_{e}^{k}(2),\ldots ,X_{e}^{k}(n)$ denote *n* extracted regions in the *k*-th layer, k∈{0,1,2,3}, $W_{1}^{k},\ldots ,W_{n}^{k}$ and $b_{1}^{k},\ldots ,b_{n}^{k}$ denote the weights and biases of the *n* fully connected networks, respectively.


$$\begin{array}{c} X_{e}^{k}:=\{X_{e}^{k}(1),X_{e}^{k}(2),\ldots ,X_{e}^{k}(n)\},\\ X_{e}^{k+1}:=\{\text{ReLU}(W_{1}^{k}X_{e}^{k}(1)+b_{1}^{k},\ldots ,\text{ReLU}(W_{n}^{k}X_{e}^{k}(n)+b_{n}^{k})\}, \end{array}$$

#### 2.4.3 Non-linear feature interaction module

We further design the non-linear feature interaction module to analyze the non-linear relationship among input features of small datasets. In particular, the second-order relation and the non-linear relation (Tanh and Sigmoid function) are used for linear and non-linear feature transformation. Given the input $X_{e}^{4}$, the calculation inside the non-linear feature interaction module is defined as: where ⊙ denotes the Hadamard product, $W_{s1},W_{s2},W_{s3},W_{s4}$ and $b_{s1},b_{s2},b_{s3},b_{s4}$ denote the weights and biases of the four fully connected network in the non-linear feature interaction module, respectively.


$$\begin{array}{c} X_{nl}^{1}:=\text{BatchNorm}((W_{s1}X_{e}^{4}+b_{s1})⊙(W_{s2}X_{e}^{4}+b_{s2})),\\ X_{nl}^{2}:=\text{Tanh}(W_{s3}X_{e}^{4}+b_{s3})⊙\text{Sigmoid}(W_{s4}X_{e}^{4}+b_{s4}),\\ X_{nl}:=\text{Dropout}(X_{nl}^{1}+X_{nl}^{2}), \end{array}$$

#### 2.4.4 Classifier

A simple fully connected layer is used as the classifier. Specifically, $X_{nl}$ is fed into the classifier, which can be described using the equation below. where W is the weight of the classifier, and $y_{\mathrm{pred}}\in R^{2}$. It is worth noting that the bias of the classifier is omitted.


$$y_{\mathrm{pred}}:=\text{Softmax}(WX_{nl}),$$

### 2.5 Training

All experiments are conducted on an Apple M1 Pro CPU and 32 GB memory. All the DL models are constructed in Python 3.9, PyG 2.1.0, and PyTorch 1.9.0. All machine-learning baseline methods, including least absolute shrinkage and selector operator (LASSO), ridge regression (RIDGE), random forest (RF), support vector machine (SVM), and eXtreme Gradient Boosting (XGBoost), are implemented based on the same Python version and scikit-learn 1.0.2.

Focal loss (Lin *et al.* 2017) and label smoothing (Müller *et al.* 2019) are combined together to customize the loss function used in training. Given the probability of classification $y_{\mathrm{pred}}$ and the true label $y_{\mathrm{true}}$, then the loss function is defined as where γ is the focal parameter, which purpose is to reduce the weight of easy-to-classify samples and increase the weight of difficult-to-classify samples so that the model can focus on the learning of samples that are difficult to be classified; $y_{s}=[0.5,0.5]$ is a 1D vector, which is used to adjust the offset degree of the positive and negative partitions; and ϵ is a customized constant, which is used to determine the proportion of the two parts in our loss function.


$$\begin{array}{c} L_{\mathrm{focal}}:=-(1-\epsilon )\times [{\left(1-y_{\mathrm{pred}}\right)}^{\gamma }\;\text{log}(y_{\mathrm{pred}})y_{\mathrm{true}}\\ +{\left(y_{\mathrm{pred}}\right)}^{\gamma }\;\text{log}(1-y_{\mathrm{pred}})(1-y_{\mathrm{true}})],\\ L_{\mathrm{smooth}}:=-\epsilon \times [{\left(1-y_{\mathrm{pred}}\right)}^{\gamma }\;\text{log}(y_{\mathrm{pred}})y_{s}\\ +{\left(y_{\mathrm{pred}}\right)}^{\gamma }\;\text{log}(1-y_{\mathrm{pred}})(1-y_{s})],\\ L:=L_{\mathrm{focal}}+L_{\mathrm{smooth}}, \end{array}$$

## 3 Results

### 3.1 Detecting chromothripsis with CNV-DAG and GECNVNet

Five train-test stratified splits are performed on the whole dataset (n=752) randomly (80% for 10-fold cross-validations, and 20% for completely isolated testing dataset). For each split, 10 trained models with the best performance based on the cross-validations are selected to test the overall performance on the completely isolated testing dataset (i.e. ensemble bagging). An intuitive figure that demonstrates the training process can be found in the Supplementary Material. The area under the precision–recall curve (AUPRC) results of the testing dataset on the five splits are reported in Table 1. The area under the receiver operating characteristic curve (AUROC) results on the testing dataset are shown in the Supplementary Material. Although AUROC is useful when evaluating general-purpose classification, AUPRC is the preferred method when classifying rare events in imbalanced datasets, especially in clinical settings.

For patients with NDMM and other bone cancers, chromothripsis occurs at the incidence rates between 20% and 30% (Stephens *et al.* 2011, Maclachlan *et al.* 2021). The ratio of positive samples in the whole dataset is similar to real imbalanced distribution, with a proportion of slightly more than 20%; it means AUPRC is the better performance metric for our task. The AUPRC of GECNVNet (0.8309 on average) exceeds other baselines significantly while maintaining a relatively high AUROC (0.9121 on average) equivalent to other baselines. To see whether the probability that GECNVNet provides can be used for prognosis inference, we further perform a survival analysis, as reported in the Supplementary Material. The results demonstrate that GECNVNet is able to infer NDMM patients’ prognosis condition via predicting the probability of chromothripsis occurrence.

Meanwhile, combining with SV Signature data and a simple MLP branch, GECNVNet can reach a new state-of-the-art performance, compared with the previous model using both CNV and SV Signature (Maclachlan *et al.* 2021) (shown in the Supplementary Material). The tuned parameters of GECNVNet are also listed in the Supplementary Material.

We also show the *t*-SNE visualization (Van der Maaten and Hinton 2008) in Supplementary Fig. S4. CNV signature data given by Maclachlan *et al.* (2021) shows a clear pattern, and patients with chromothripsis can be easily divided into two parts linearly; it explains why linear models work well on CNV signature data. Compared to the CNV signatures, the original CNV feature data (the original feature input of the 28 CNV features) do not have any linearly separable pattern, emphasizing the ability of the classifier to extract complex features. Supplementary Figure S4C–E shows the *t*-SNE visualization of the feature representations given by three main modules of GECNVNet. The graph embedding module is able to give a robust feature embedding representation, as depicted in Supplementary Fig. S4C, where the feature pattern is not random. The local feature extraction module can extract the data linearly, and feature representations of samples with chromothripsis are projected to one side in Supplementary Fig. S4D. The samples with chromothripsis events are gathered in a small region for downstream classification after the non-linear feature interaction module, as shown in Supplementary Fig. S4E. Overall, the three main modules in GECNVNet convert the random pattern of CNV features to distinguishable patterns, thereby achieving higher performance than other baselines.

### 3.2 Genomic insights from the CNV-DAG

In the derived CNV-DAG, the source vertex can affect other vertices through intermediate variables or a direct edge (Guo *et al.* 2020). Therefore, in order to explore the impact of the DAG structure on the whole graph from the source, we examine a group of representative edges from the source vertex (i.e. BAND) to three other vertices (i.e. COUNT, SIZE, and OSCI) in the CNV-DAG (excluding virtual vertex and virtual edges) for analysis. The node BAND represents the global CN breakpoints and alteration per chromosome (Korbel and Campbell 2013). The higher existence of CN breakpoints on a single chromosome indicates a higher probability of DNA rearrangements (Stephens *et al.* 2011). Hence, the local CN breakpoints in unit chromosome length (COUNT) are possibly increased. Meanwhile, because the node OSCI is generated by evaluating the total length of oscillating CN segments, more CN breakpoints on a single chromosome would likely lead to longer consecutive oscillating fragments (Korbel and Campbell 2013). Moreover, there is a high probability that additional CN breakpoints on a chromosome bring extra CN segments, affecting the CN segment size (SIZE). However, the specific relevance is difficult to be identified due to the stochasticity of DNA rearrangements and chromothripsis occurrence. Additionally, the rearrangement of fragments and amplification of double-minute chromosomes will also lead to various CN segmental sizes (Carroll *et al.* 1988, Stephens *et al.* 2011). Therefore, we indicate that the edge BAND→SIZE is less significant than the edges BAND→COUNT and BAND→OSCI.

**Figure 2.**
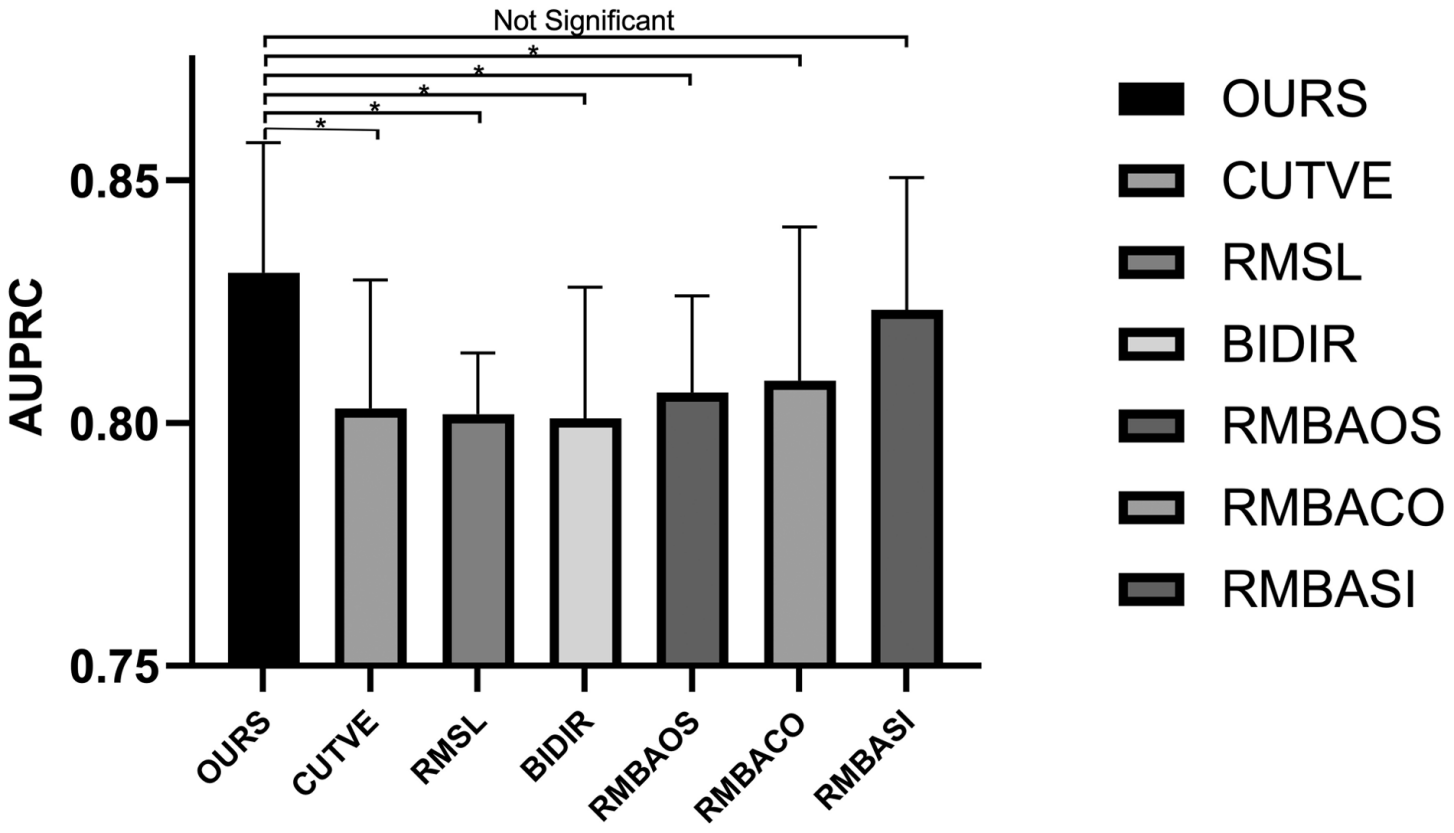
AUPRC of GECNVNet under different conditions. CUTVE: the virtual edges to the virtual node are removed. RMSL: self-loops of the actual nodes are removed. BIDIR: changing the directed graph to undirected graph. RMBAOS: the actual edge from BAND to OSCI is removed. RMBACO: the actual edge from BAND to COUNT is removed. RMBASI: the actual edge from BAND to SIZE is removed. *P-value of the Wilcoxon signed-rank test of AUPRC < .05

Ablation experiments on GECNVNet are conducted to verify the deductions above. Apart from the edges mentioned, the following conditions are also included in the ablation experiment: removing all the virtual edges, removing self-loops, and changing the directed graph to an undirected graph. The AUPRC results of the ablation experiment are shown in Fig. 2. The AUROC results are shown in the Supplementary Material. We also compare the performance using the original causal DAG shown in Supplementary Fig. S1. In Fig. 2, the AUPRC scores of GECNVNet under different conditions fluctuate a lot. This means the ability to classify the positive samples is affected. Three conditions affect the performance of GECNVNet most (CUTVE, RMSL, and BIDIR) because more than one edge are removed. Under the conditions of RMBAOS and RMBACO, the AUPRC decreases more than that under the RMBASI. The results verify the indication that the edges from BAND to OSCI and COUNT are more significant than the edge from BAND to SIZE, as we mentioned above.

**Figure 3.**
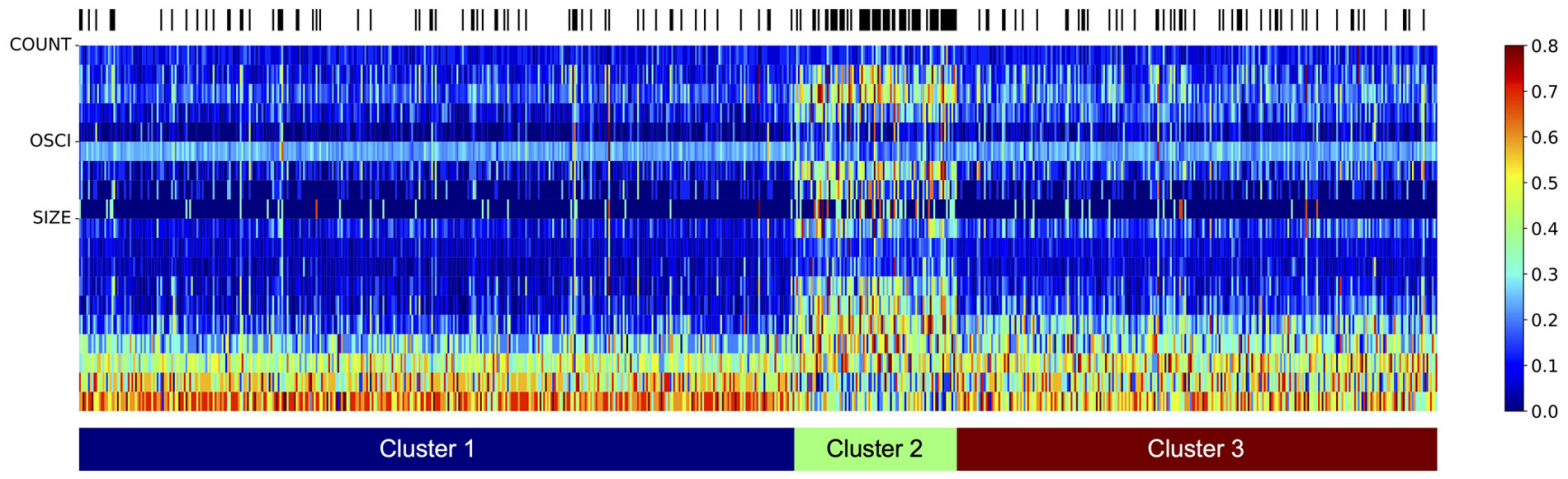
A heatmap of COUNT, OSCI, and SIZE features. Samples are re-ordered and grouped according to the clustering result of BAND features. The presence of chromothripsis is annotated in black in the top bar

Furthermore, to support that the derived edges in CNV-DAG are reasonable, a clustering analysis is also conducted. *K*-means clustering is firstly run over BAND features to split the samples into k=3 clusters, where the silhouette coefficient is leveraged as the metric to evaluate the effect of clustering and determine the optimal *k* value. According to the clustering result, we further show the heatmap of COUNT, OSCI, and SIZE features in Fig. 3. In Fig. 3, the samples in Cluster 2 have a completely distinguished pattern compared to the samples in the other two clusters, with more occurrences of all three types of CNV features. Moreover, compared to samples in Cluster 1, samples in Cluster 3 have slightly more COUNT and OSCI values. The distribution of SIZE in Cluster 3 is also different from that in Cluster 1, with more medium SIZE values and less large SIZE values. Most importantly, the distribution of chromothripsis occurrence of samples grouped in Cluster 2 is dense, which means that higher COUNT and OSCI values may be a signal of the chromothripsis event. Overall, the clustering results can support that the edges from the source vertex to the other three vertices in CNV-DAG are reasonable.

### 3.3 Feature importance analysis

**Figure 4.**
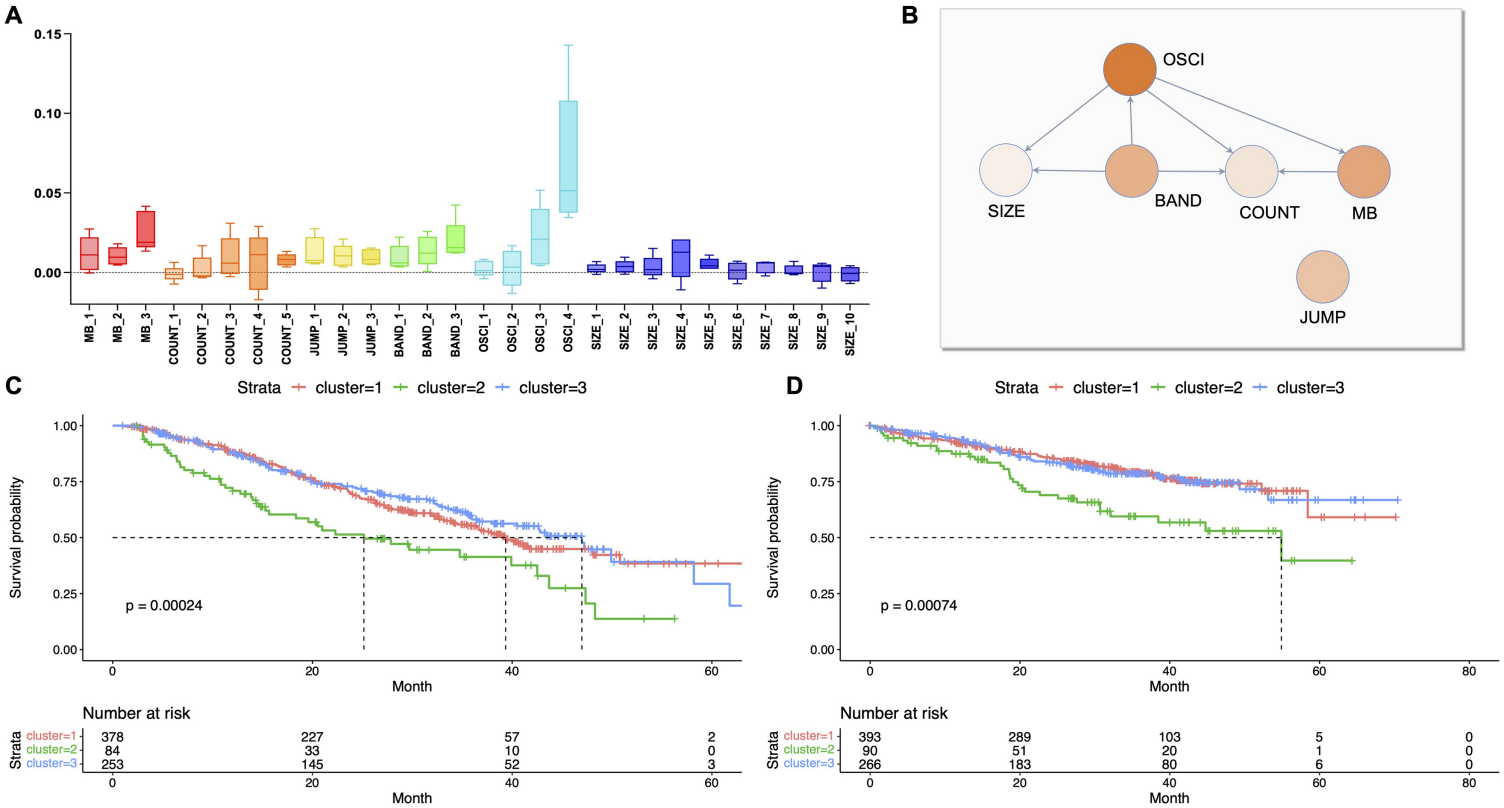
Feature importance analysis. (A) The feature importance comparison of the 28 CNV features on five splits calculated by GradientShap. GradientShap values on five splits are presented as boxplot. (B) The feature importance visulization on the CNV-DAG, where higher feature importance is shown with higher opacity. For clarity, virtual nodes and virtual edges are not shown. (C and D) Kaplan–Meier curve of PFS and OS probability according to the clustering result in Section 3.3 (Cluster 1 and Cluster 3: the top two overlapped lines, Cluster 2: the lowest separate line). The *P*-values are calculated according to a two-sided log-rank test

In order to make the black-box model interpretable and verify the effect of high COUNT and OSCI values on chromothripsis, the GradientShap method is leveraged to examine the importance of the 28 CNV features (Lundberg and Lee 2017). GradientShap is designed to find each feature’s marginal contribution to the predictive class that the neural network outputs (i.e. SHAP value). The features’ importance is calculated using GradientShap on the trained models of the five splits, and the results are shown in Fig. 4. OSCI_4 has the highest impact on the positive classification result, which means the large number of contiguous CN segments alternating between two CN states is considered a strong signal of chromothripsis occurrence. However, compared to OSCI features, COUNT features may not have much effect on chromothripsis. All SIZE features have minimal impact on the classification result, which indicates that CN segment size is not related to chromothripsis. Moreover, MB features have higher importance values than BAND features. This phenomenon possibly explains that local CN breakpoints are more relevant in chromothripsis detection than CN breakpoints in a global chromosome arm. Most interestingly, we observe that the nodes with higher out-degree in the CNV-DAG have relatively high feature importances (e.g. BAND and OSCI). In contrast, nodes with higher in-degree and without out-degree in the CNV-DAG have lower feature importances (e.g. COUNT and SIZE), as shown in Fig. 4B. Such a phenomenon is likely because a slight change of BAND and OSCI in the CNV-DAG can affect multiple other nodes, thus leading to chromothripsis. In contrast, because in the CNV-DAG, node SIZE and COUNT have an out-degree of 0, the change of COUNT and SIZE may not affect other features. Therefore, they have lower feature importance.

Additionally, we notice that samples in Cluster 2 have higher OSCI values in Section 3.2. In order to explore whether higher OSCI values may be associated with poor clinical outcomes, a survival analysis of the three clusters is conducted according to the clustering result in Section 3.2. The Kaplan–Meier curves of PFS and OS according to the clusters shown in Fig. 4C and D demonstrate that the NDMM patients in Cluster 2 who have longer OSCI length potentially have shorter PFS and OS. At the same time, the clinical outcomes of the patients in Clusters 1 and 3 are not significantly different; it means the changes in SIZE features may not be associated with poor prognostic effects, as shown in Fig. 4C and D.

## 4 Discussion

Although intensive research in chromothripsis has been conducted for almost ten years, the reasons why this phenomenon occurs are still partially transparent (Kolb and Ernst 2021). At the same time, reliable, accurate, and rapid chromothripsis detection remains a subject worth exploring (Maclachlan *et al.* 2021). In this study, a novel approach is presented to detect chromothripsis by leveraging structure learning and a novel neural network architecture. Survival analysis also supports that the NDMM patients with chromothripsis detected by GECNVNet exhibit distinct prognostic characteristics (i.e. shorter PFS and OS).

In our methodology, structure learning enables us to elucidate the intrinsic causal relationships between different CNV features, as captured by the derived CNV-DAG. The results of the ablation experiments and clustering analysis further support the CNV-DAG is reasonable, and also verify our possible deductions in the derived CNV-DAG that specific edges (e.g. BAND→COUNT and BAND→OSCI) are more important. Meanwhile, from the feature importance analysis, we notice that the large number of long oscillating CN segment chains (OSCI) is a strong indicator of chromothripsis. Interestingly, we also observe that, in the CNV-DAG, the nodes with high out-degrees have higher importance compared to the nodes with high in-degrees and zero out-degree. This phenomenon may provide interesting perspectives on chromothripsis.

Current approaches for identifying chromothripsis require both CNV and SV data (Cortés-Ciriano *et al.* 2020, Ashby *et al.* 2022). Our study provides an explainable DL way to detect chromothripsis without SV. Moreover, since chromothripsis is emerging as one of the most robust indicators for predicting the development of myeloma precursor condition into MM, as well as shorter PFS and OS in NDMM patients (Maclachlan *et al.* 2021), it can contribute to the development of earlier personal diagnosis and prognosis scoring system. Furthermore, our process to form the CNV-DAG can be transferred and applied to other types of cancer, potentially providing new insights into the molecular mechanisms of other cancers. In the future, we will migrate the methodology proposed in this study to see whether structure learning can bring other exciting insights.

## Supplementary Material

btad422_Supplementary_Data

## Data Availability

The data underlying this article are available in Github at https://github.com/luvyfdawnYu/CNV_chromothripsis.
