# Supplementary material for: Chromothripsis detection with multiple myeloma patients based on deep graph learning
Source: Bioinformatics. 2023 Jul 3;39(7):btad422. doi: 10.1093/bioinformatics/btad422 (PMC10343948; doi:10.1093/bioinformatics/btad422)
Supplement: btad422_Supplementary_Data [file btad422_supplementary_data.pdf]

Supplementary Information

Algorithm 1 The training process of GECNVNet

Data: The whole dataset $D = \{\mathbf{x}^{(n)}, y^{(n)}\}_{n=1}^{N}$ with $N = 752$ samples

Result: The ensemble chromothripsis detection probability of GECNVNets $\hat{\mathbf{Y}}_{test}$ and the evaluation metric $\mathcal{M}$

Perform a random stratified split on the whole dataset D to obtain a training set D_{train} and an isolated test set D_{test}, where $|D_{test}| = \frac{N}{5}$.

Separate the training set D_{train} into 10 folds, denoted as $\{D_{train}^{(1)}, D_{train}^{(2)}, ..., D_{train}^{(10)}\}$, where $\forall i, |D_{train}^{(i)}| = \frac{|D_{train}|}{10}$.

```
// Perform a 10-fold cross-validation on the training set
for i = 1 to 10 do
    Select the {D_train^(¬i)} as the training set, and the D_train^(i) as the validation set.
    Initialize a GECNVNet M^(i) with the parameters θ_0^(i)
    for each epoch do
        for each mini-batch B^(i) = {x_n, y_n}_{n=1}^{batch_size} ∈ D_train^(¬i) do
            // Compute the gradients of the loss function L with respect to θ^(i) based on mini-batch B
            Y_pred = {M^(i)(x_n) | x_n ∈ B^(i)}
            Y_true = {y_n | y_n ∈ B^(i)}
            loss = L(Y_true, Y_pred)
            g = ∇_θ loss
            // Update the parameters of the model M^(i) according to the learning rate α
            θ^(i) ← θ^(i) − αg
            Evaluate the model M^(i) on the validation set {D_train^(i)}.
            if the performance of current model is better than previous best one then
                Save current parameters θ^(i)
            end
        end
    end
end
// Initialize the obtained best parameters on 10 folds
Initialize 10 GECNVNets {M^(1), M^(2), ..., M^(10)} with parameters {θ^(1), θ^(2), ..., θ^(10)}
Y_test = {y_n | y_n ∈ D_test}
for i = 1 to 10 do
    Compute the detection probability Ŷ_test^(i) given by the i-th model M^(i) on the test set D_test
end
// Make ensemble decision
Ŷ_test = Σ_{i=1}^{10} Ŷ_test^(i) / 10
Compute the evaluation metric M using Y_test and Ŷ_test
```

Description and threshold of the CNV features

The description and threshold of the CNV features are listed in the supplementary Table S1. Here we take the MB as an example to show how the trainable graph is established. MB denotes the number of CN breakpoints per 10 Mb. In our case we have three sub-features of MB divided by the thresholds given by the *mclust* package, namely, MB_1, MB_2, MB_3. Then we model the MB as a node in the graph, with a feature vector of [MB_1, MB_2, MB_3]. We do this process for all types of CNV Feature and connect the nodes with the edges in the original causal DAG shown in supplementary Figure S1. Before being fed into the neural network, the node features are all padded with zeros for feature alignment and the convenience of representation learning.

Table S1. Description and threshold of the CNV features

CNV Feature	Count Threshold
MB_1	3
MB_2	6
MB_3	31
COUNT_1	0
COUNT_2	1
COUNT_3	2
COUNT_4	3
COUNT_5	9
JUMP_1	1
JUMP_2	3
JUMP_3	8
BAND_1	5
BAND_2	17
BAND_3	60
OSCI_1	1
OSCI_2	4
OSCI_3	9
OSCI_4	38
SIZE_1	165300
SIZE_2	502740
SIZE_3	1588400
SIZE_4	7113100
SIZE_5	21801700
SIZE_6	53962800
SIZE_7	67587881
SIZE_8	105333496
SIZE_9	142322700
SIZE_10	249137896

The original derived causal graph of the CNV Features

The visualization of the original derived causal graph is shown in supplementary Figure S1.

The training process and the data spilt

An intuitive figure that demonstrates the training process is shown in supplementary Figure S2. The detailed training process of GECNVNet is shown in supplementary Algorithm 1.

The tuned hyperparameters of GECNVNet

The tuned hyperparameters of GECNVNet is shown in supplementary Table S2.

The experimental result of AUROC on five different splits

The experimental result of AUROC on five different splits is shown in supplementary Table S3.

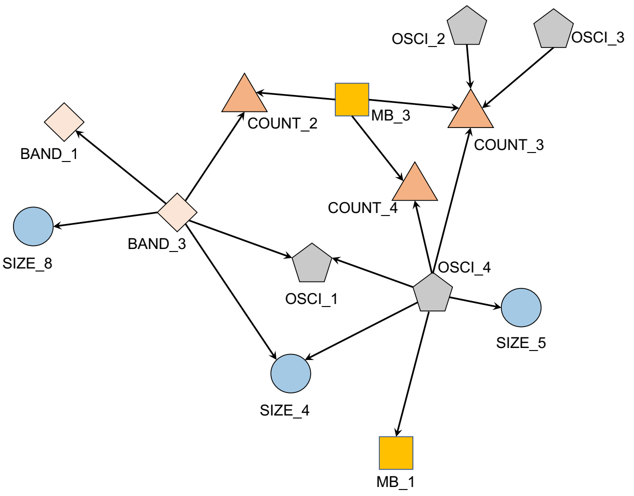

Fig. S1. There are twenty-eight nodes in the original output causal DAG. The nodes in the DAG denote the CNV features. In order to find the most significant relationships between these nodes, the edges with low weights (i.e., edges with weight < 2) are deleted. The description of the twenty-eight CNV features nodes are reported in supplementary Table S1.

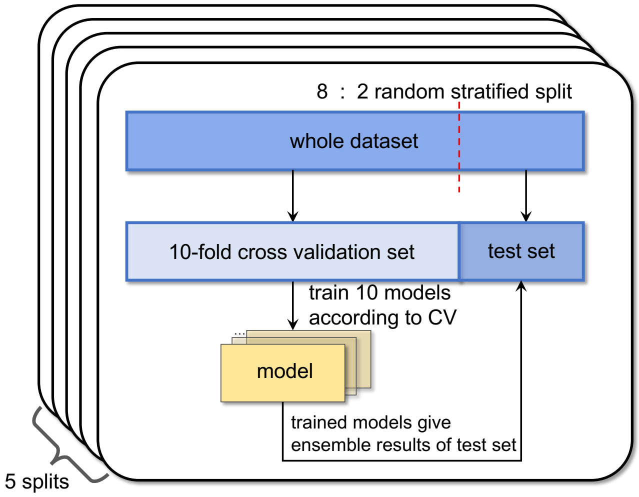

Fig. S2. The experiment process of a given model. Our experiment process involved several steps. First, we perform a random stratified split on the whole dataset to obtain a training set and a test set. Next, we apply a 10–fold cross validation to train the model and get ten independent trained model. Subsequently, we use the trained models to detect the chromothripsis on the test set and employ a bagging technique to obtain a more robust joint decision (i.e., ensemble learning) of these models. To assess the robustness of the models, we repeat the above process five times, resulting in detection results for five splits (i.e., Split1–5 in Table 1).

The AUPRC performance gain and loss compared to average performance

The AUPRC performance gains and losses compared to average performance of all models are shown in supplementary Figure S3.

Prognosis inference of the NDMM patients

To further show that the chromothripsis detected by GECNVNet is reliable from a clinical perspective and obtain a reasonable estimate of the clinical outcome, we conduct a survival analysis on the NDMM patients. The Kaplan – Meier curves of PFS and OS probability are computed and drawn according to the prognosis profile. The patients are divided into two groups according to the classification probability given by the GECNVNet. According to Maclachlan et al. (Maclachlan *et al.*, 2021), in order to get better classification, those with a probability of more than 0.6 are defined as the high-risk group, while the others are defined as the low-risk group. The results are shown in supplementary Figure S4. The Kaplan–Meier curves in supplementary Figure S4A and supplementary Figure S4B show a significant difference in the probability of PFS ($p =$ 0.00034) and the probability of OS ($p < 0.0001$) between the group with high risk and the group with low risk. Survival analysis on these patients demonstrates that the probability of the chromothripsis event provided by GECNVNet is a strong predictor of a shorter PFS and OS. A backward step-wise Cox regression multivariate analysis is also performed to select the most critical features from highly correlated genomic risk factors (Maclachlan *et al.*, 2021). The group classification shows a significant association with shorter PFS and OS in the high-risk group after controlling for other variables in the model, producing a hazard ratio (HR) of 1.70 (95% CI 1.26 – 2.31, p=0.001), and 1.90 (95% CI 1.30 – 2.77, p=0.001), respectively. The above analysis demonstrates that GECNVNet is able to infer NDMM patients' prognosis condition via predicting the probability of chromothripsis occurrence.

Table S2. Hyperparameters of GECNVNet

Module/Hyperparameter Name	Dimension/Value
Input	(7×10)
Graph Transformer layer 1	$(7 \times 10) \to (7 \times 16)$
Graph Transformer layer 2	$(7 \times 16) \to (7 \times 32)$
Bilinear pooling	$(7 \times 32) \to (1024)$
Local feature extraction layer–1	$(1024) \to (768)$
Local feature extraction layer–2	$(768) \to (512)$
Local feature extraction layer–3	$(512) \to (256)$
Local feature extraction layer–4	$(256) \to (128)$
Nonlinear feature interaction module	$(128) \to (128)$
Classifier	$(128) \to (2)$
Learning rate	1e-2
Batch size	128
Epochs	120

The effect of SV data

SVs include translocations, insertions, and deletions, which are closely related to chromothripsis (Mahmoud *et al.*, 2019). Therefore, combining SV data with other genomic alterations, such as copy number variations (CNVs), may result in more effective detection of chromothripsis (Rustad *et al.*, 2020). We replicate the method proposed by Maclachlan et al (Maclachlan *et al.*, 2021), which combines both SV Signature and CNV Signature. Our results show that the incorporation of SV data improves chromothripsis detection a lot, as evidenced by an increase in the average area under the precision-recall curve (AUPRC) from 0.8001 (LASSO + CNV Signature) to 0.9170 (LASSO + CNV Signature and SV Signature). Moreover, we extend GECNVNet with a four-layer multi-layer perceptron that feeds on SV Signature data (GECNVNet and 4-layer MLP

Table S3. The experimental result of AUROC on five different splits.

		Split1	Split2	Split3	Split4	Split5	AUROC (AVG±STD)
CNV Signature	LASSO (Maclachlan *et al.*, 2021)	0.9464	0.9145	0.8872	0.9140	0.9056	0.9135±0.021
	RIDGE	0.9449	0.9140	0.8836	0.9106	0.9039	0.9114±0.022
	SVM(RBF)	0.9147	0.8940	0.8399	0.9060	0.8940	0.8897±0.029
	SVM(linear)	0.9302	0.9058	0.8597	0.9029	0.9140	0.9025±0.026
	RF	0.9192	0.9121	0.8807	0.9134	0.8900	0.9031±0.017
	XGBoost	0.8925	0.9058	0.8804	0.9089	0.8983	0.8972±0.011
CNV Feature	LASSO	0.9099	0.9005	0.8899	0.9058	0.8732	0.8959±0.015
	RIDGE	0.8959	0.9012	0.8877	0.9010	0.8681	0.8908±0.014
	SVM(RBF)	0.9215	0.8908	0.8739	0.9210	0.8877	0.8990±0.021
	SVM(linear)	0.8862	0.8845	0.8829	0.9123	0.8802	0.8892±0.013
	RF	0.9407	0.8987	0.8948	0.9168	0.9023	0.9107±0.019
	XGBoost	0.9126	0.8826	0.8713	0.9017	0.9000	0.8936±0.016
	MLP	0.9379	0.8998	0.8879	0.8529	0.9053	0.8968±0.031
	CNN	0.9130	0.9114	0.9087	0.9097	0.8594	0.9004±0.023
	Graph Transformer (Shi *et al.*, 2020)	0.9331	0.8884	0.8829	0.8901	0.8671	0.8923±0.026
	GCN (Kipf and Welling, 2016)	0.9278	0.8867	0.8662	0.8773	0.8657	0.8847±0.026
	GAT (Veličković *et al.*, 2017)	0.9283	0.8715	0.8698	0.8848	0.8691	0.8847±0.025
	Transformer (Vaswani *et al.*, 2017)	0.9135	0.8983	0.8771	0.9157	0.8807	0.8970±0.018
	GECNVNet(Ours)	0.9408	0.9128	0.8908	0.9244	0.8915	0.9121±0.022

CNV Signature: the data input is processed using HDP mixture model as Maclachlan et al. (Maclachlan *et al.*, 2021). CNV Feature: the data input is the original 28 categories CNV feature, SVM(RBF): SVM with Gaussian Radial Basis function kernel, SVM(linear): SVM with linear function kernel, MLP: a four-layer multi-layer perceptron neural network, CNN: a four-layer convolutional neural network, Graph Transformer: a four-layer Graph Transformer neural netowork, GCN: a four-layer graph convolutional neural network, GAT: a four-layer graph attention neural network, Transformer: a four-layer Transformer encoder neural network.

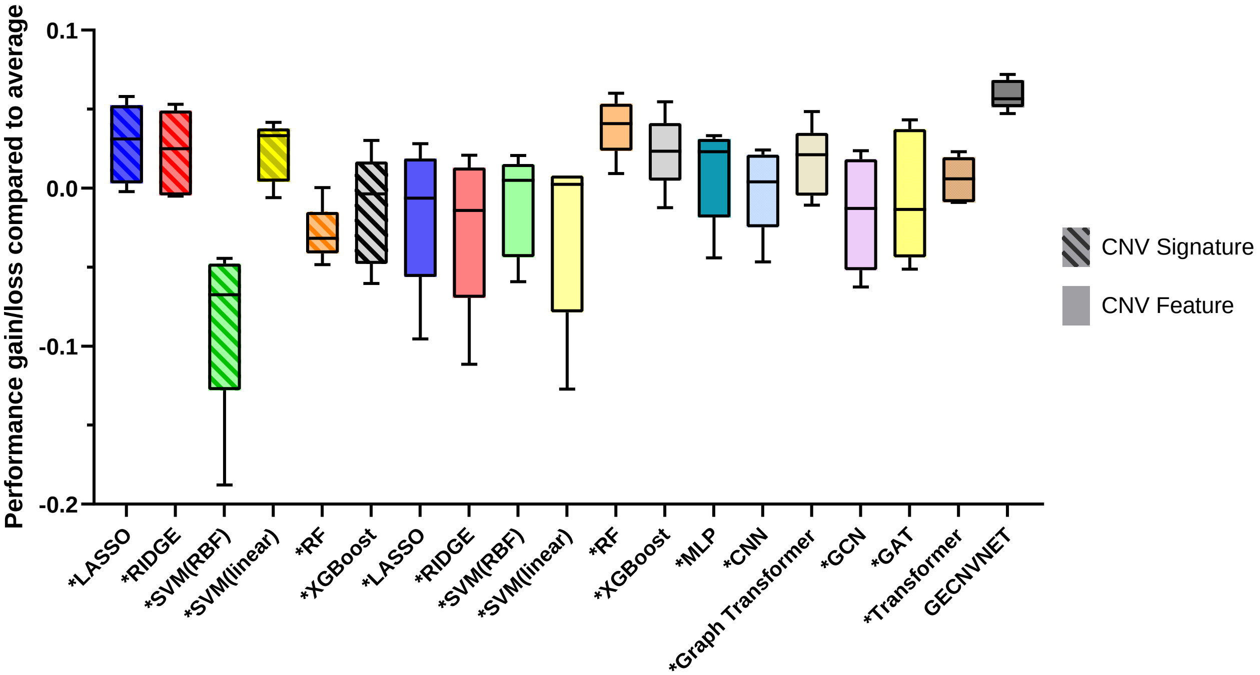

Fig. S3. The AUPRC performance gain/loss compared to average. For each split, we calculate the performance gain or loss of all methods compared to the average performance on this split according to Table 1 in the main text. The boxes with dashed line indicate the data input is CNV Signature, while the pure-coloured boxes indicate the data input is CNV Feature.

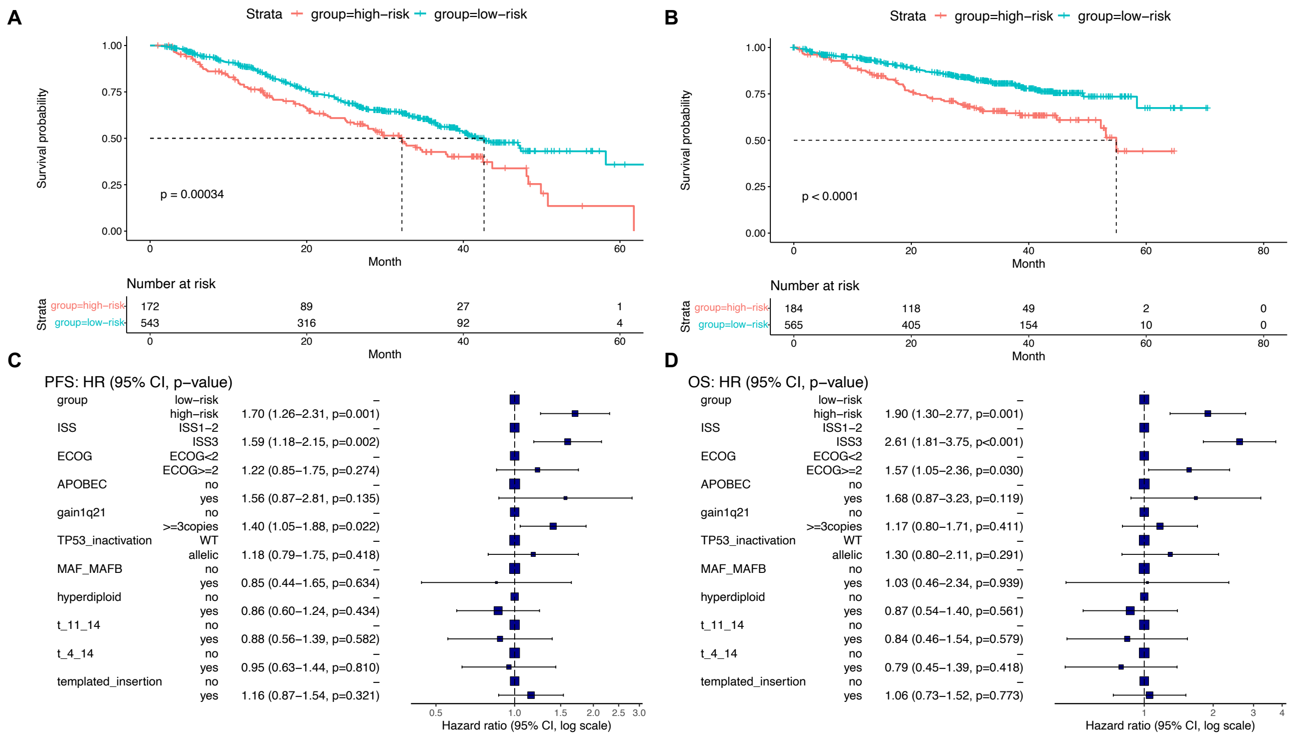

Fig. S4. The results of survival analysis shows that the groups divided by GECNVNet are predictive of clinical outcomes of NDMM patients. (A, B): Kaplan – Meier curve of PFS and OS probability according to high–risk (blue) or low–risk (red) groups. The p-values are calculated according to a 2–sided log-rank test. (C, D): Multivariate analysis of the effect of group classification on PFS and OS for International Staging Score (ISS), Eastern Cooperative Oncology Group (ECOG) score, APOBEC mutational activity, gain/amplification of 1q21 (gain1q21), TP53 inactivation, translocations involving MAF/MAFB (MAF_MAFB), hyperdiploid, translocations of 11–th and 14–th chromosome(t_11_14), translocations of 4–th and 14–th chromosome(t_4_14), and templated insertions. Multivariate analysis was performed by the Cox proportional hazards model with p-values according to a 2-sided Wald test. Data is presented as median values ± 95% confidence interval.

+ CNV Feature and SV Signature), which further improved the AUPRC performance to 0.9225, surpassing the baseline method (Maclachlan *et al.*, 2021). The integration of SV suggests that SV is a strong factor that improve the detection results in both GECNVNet and the baseline (Maclachlan *et al.*, 2021), resulting a slight difference in the performance between GECNVNet and the model proposed by Maclachlan et al (Maclachlan *et al.*, 2021).

Furthermore, we conduct a Chi-Square test to compare the high-risk and low-risk patient groups identified by GECNVNet using CNV Feature only and the baseline method that integrates both CNV and SV signatures. The results indicate that there is no statistically significant difference in the ability to identify high-risk MM patients between the proposed model and the baseline, with a p-value of 0.44. Hence, our proposed model still has considerable performance and clinical value in the absence of SV data, which is difficult to be extracted.

t-SNE visualization

The t-SNE visualization of the feature representations given by the modules in GECNVNet is shown in supplementary Figure S5.

AUROC results of the ablation experiment

The AUROC results of the ablation experiment is shown in supplementary Figure S6 and supplementary Table S4. In supplementary Table S4, using the original causal DAG, the performance has a drop on every split. We indicate this is because there are too many isolated nodes in the graph, and the message passing mechanism could not be utilized in an efficient manner. Through aggregating the edges, we can maintain the causal relationship in the original causal DAG and insert more information into the DAG, thereby achieving higher performance.

The AUROC performance comparison on fully-balanced test set

Here we downsample all the test sets in five different splits to achieve a fully balanced case-control ratio and use AUROC as the evaluation metric. The results are shown in supplementary Figure S7. Our GECNVNet still maintains a high AUROC on five splits, and exceeds all other baselines.

References

Kipf, T. N. and Welling, M. (2016). Semi-supervised classification with graph convolutional networks. *arXiv preprint arXiv:1609.02907*.

Maclachlan, K. H. *et al.* (2021). Copy number signatures predict chromothripsis and clinical outcomes in newly diagnosed multiple myeloma. *Nature communications*, **12**(1), 1–11.

Mahmoud, M. *et al.* (2019). Structural variant calling: the long and the short of it. *Genome biology*, **20**(1), 1–14.

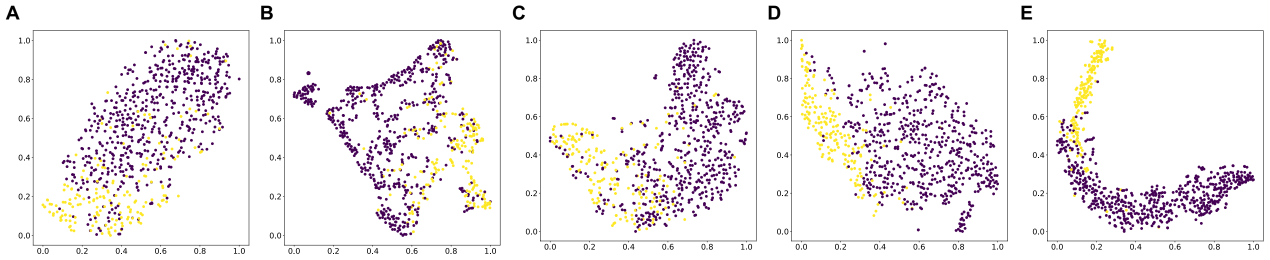

Fig. S5. t-SNE visualization of CNV data at different stages. The yellow points denote the patients who have chromothripsis, while the dark blue ones denote the non-chromothripsis patients. (A): t-SNE visualization of the CNV feature shows a random pattern. (B): t-SNE visualization of the CNV signature shows a different pattern. (C-E): t-SNE visualization of the output feature representations after the graph embedding module, the local feature extraction module, and the nonlinear feature interaction module, respectively.

Table S4. The AUPRC comparison between using the original causal graph (shown in supplementary Figure S1) and the aggregated one (shown in Figure 1).

Methods	Split1	Split2	Split3	Split4	Split5	AUPRC (AVG±STD)
GECNVNet w/ original causal graph	0.8461	0.7837	0.8011	0.7728	0.7928	0.7993±0.028
GECNVNet w/ aggregated causal graph	**0.8787**	**0.8176**	**0.8202**	**0.8211**	**0.8171**	**0.8309±0.027**

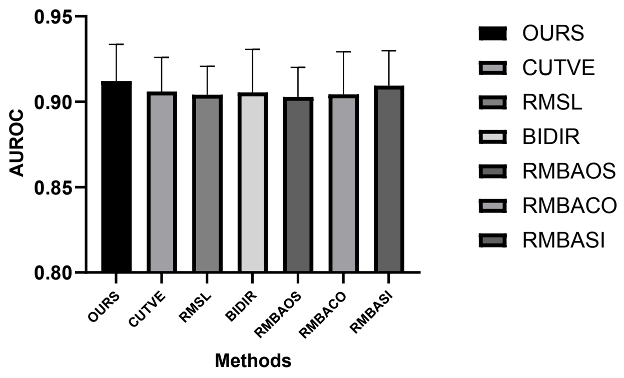

Fig. S6. AUROC of GECNVNet under different conditions. CUTVE: the virutual edges to the virtual node are removed. RMSL: self-loops of the actual nodes are removed. BIDIR: changing the directed graph to undirected graph. RMBAOS: the acutal edge from BAND to OSCI is removed. RMBACO: the actual edge from BAND to COUNT is removed. RMBASI: the actual edge from BAND to SIZE is removed.

Rustad, E. H. *et al.* (2020). Revealing the impact of structural variants in multiple myeloma. *Blood cancer discovery*, **1**(3), 258–273.

Shi, Y. *et al.* (2020). Masked label prediction: Unified message passing model for semi-supervised classification. *arXiv preprint arXiv:2009.03509*.

Vaswani, A. *et al.* (2017). Attention is all you need. *Advances in neural information processing systems*, **30**.

Veličković, P. *et al.* (2017). Graph attention networks. *arXiv preprint arXiv:1710.10903*.

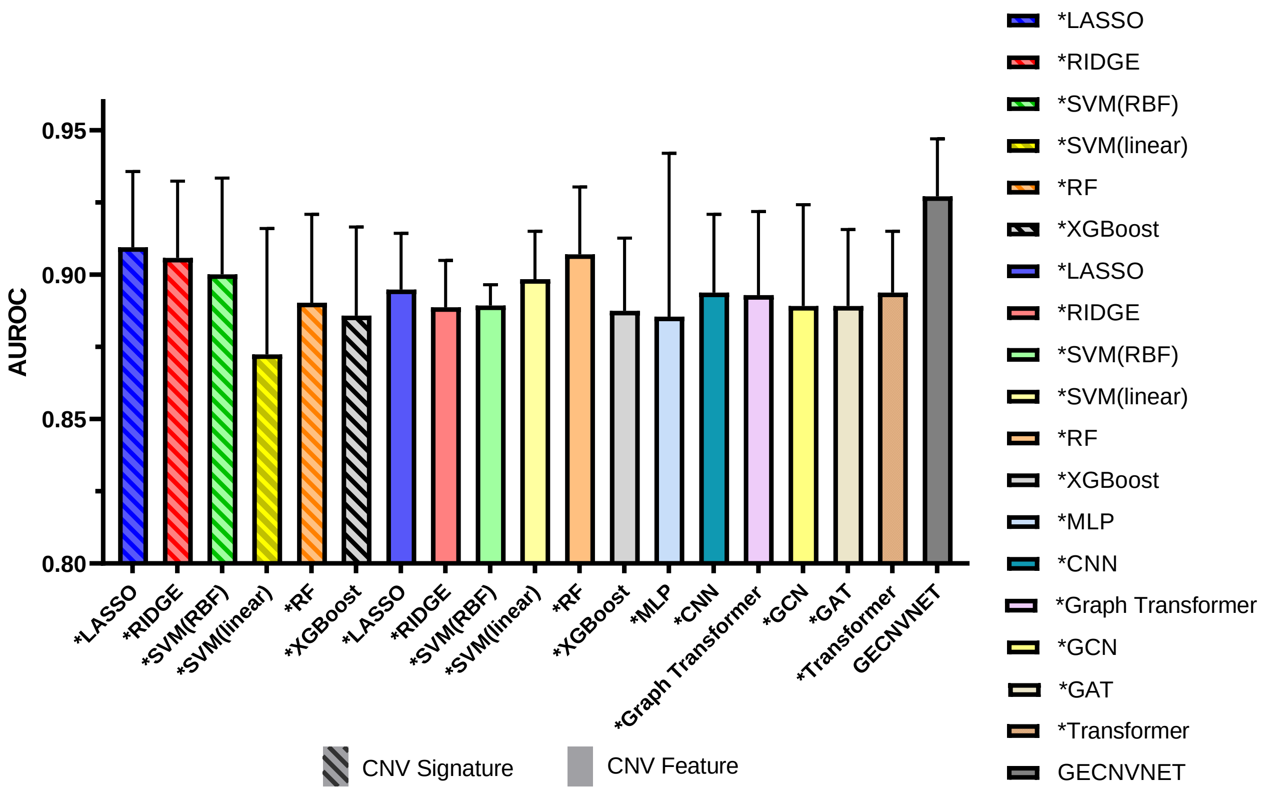

Fig. S7. The experimental result of AUROC on five downsampled test sets. CNV Signature: the data input is processed using HDP mixture model as Maclachlan et al. (Maclachlan *et al.*, 2021). CNV Feature: the data input is the original 28 categories CNV feature, SVM(RBF): SVM with Gaussian Radial Basis function kernel, SVM(linear): SVM with linear function kernel, MLP: a four-layer multi-layer perceptron neural network, CNN: a four-layer convolutional neural network, Graph Transformer: a four-layer Graph Transformer neural netowork, GCN: a four-layer graph convolutional neural network, GAT: a four-layer graph attention neural network, Transformer: a four-layer Transformer encoder neural network, *: p-value of the Wilcoxon signed-rank test of AUROC < 0.05.
